# *Helicobacter cinaedi* bacterium association with atherosclerosis and other diseases

**DOI:** 10.3389/fmicb.2024.1371717

**Published:** 2024-04-08

**Authors:** Alice K. Voronina, Georgij P. Arapidi

**Affiliations:** ^1^Lopukhin Federal Research and Clinical Center of Physical-Chemical Medicine of Federal Medical Biological Agency, Moscow, Russia; ^2^Shemyakin-Ovchinnikov Institute of Bioorganic Chemistry of the Russian Academy of Sciences, Moscow, Russia

**Keywords:** *Helicobacter cinaedi*, atheroscelorsis, foam cell, macrophage, cardiovascular diseases

## Abstract

*Helicobacter* is a genus of spiral-shaped Gram-negative enterohepatic bacteria whose members are capable of causing bacteremia in humans. One of the poorly studied members of this genus is the bacterium *Helicobacter cinaedi*. This microorganism was first isolated from human fecal samples in 1984. Although it was long considered to be associated with only immunocompromised patients, more evidence in recent years has implicated *H. cinaedi* in causing serious pathologies in immunocompetent populations. In addition, *H. cinaedi* is also reported to be associated with a few chronic or severe illnesses, such as atherosclerosis, which in turn can lead to the development of other cardiovascular pathologies: one of the leading causes of mortality worldwide. *Helicobacter cinaedi* often goes unnoticed in standard diagnostic methods due to its slow growth under microaerobic conditions. This often leads to significant underdetection and hence undermines the role of this bacterium in the pathogenesis of various diseases and the extent of its spread in humans. In this review, we have compiled information on pathologies associated with *H. cinaedi*, the occurrence of the bacterium in humans and animals, and the latest developments in diagnosing the bacterium and treating associated diseases.

## 1 Introduction

Cardiovascular diseases (CVD) have been the leading cause of mortality worldwide for the last decades, including the Russian Federation (WHO website https://www.who.int/health-topics/cardiovascular-diseases). In turn, most cardiovascular diseases (e.g., coronary heart disease, myocardial infarction, and stroke) occur due to atherosclerotic lesions of blood vessel walls, leading to inflammation and further development of pathology (Björkegren and Lusis, 2022). To date, the mechanisms of lipoprotein metabolism disorders causing atherosclerosis are known, but the exact causes have not yet been established. The main mechanism of atherosclerosis development include disruption of the normal metabolism of lipids and proteins in the inner layer of arteries, or intima, which results in cholesterol plaque formation in the vessel lumen (Fan and Watanabe, 2022).

*Chlamydia pneumoniae* was considered for a long time as a microorganism associated with the development of atherosclerosis (Shor et al., 1992). However, it has been demonstrated that although *C. pneumoniae* is widely distributed in coronary atherosclerotic plaques of patients undergoing coronary atherectomy, the degree of *C. pneumoniae* infection is not associated with plaque instability or restenosis after atherectomy (Otani et al., 2022). For *Helicobacter pylori*, an indirect involvement in the development of atherosclerosis has been shown. The chronic inflammatory process induced by the bacterium is thought to be an indirect mechanism of endothelial cell damage contributing to the development of atherosclerosis (Chmiela et al., 2015; Krupa et al., 2021). Another hypothesis suggests that the interaction of *H. pylori* with other members of the internal microflora may lead to dysbiosis, resulting in increased levels of trimethylamine N-oxide, which is an inducer of endothelial damage, in serum (Francisco, 2022). Therefore, the role of microflora (including bacteria associated with atherosclerotic plaques) in the development of atherosclerosis is still not fully understood.

Also there is much evidence that the internal microbiota of the human organism may indirectly contribute to the course of atherosclerotic processes (Verhaar et al., 2020). The gut microbiota of patients with symptomatic atherosclerosis has been shown to have a higher abundance of *Collinsella*, *Enterobacteriaceae*, *Streptococcaceae*, and *Klebsiella* and a lower abundance of short-chain fatty acid-producing bacteria such as *Eubacterium*, *Roseburia*, and *Ruminococcaceae* compared to healthy individuals (Jie et al., 2017; Liu et al., 2019). It was hypothesized that pathogenic bacteria originating from the oral or gut microbiome make vessel walls more prone to plaque formation by directly infecting the vessel wall or inducing an autoimmune inflammatory response through molecular mimicry (Epstein et al., 2000; Rosenfeld and Campbell, 2011). The analysis of plaque contents has detected bacteria of the genus *Streptococcus*, *Pseudomonas*, *Klebsiella*, *Veillonella*, as well as microbes such as *Chlamydia pneumoniae* or *Helicobacter pylori* (Ott et al., 2006; Koren et al., 2011; Lanter et al., 2014; Mitra et al., 2015).

According to one hypothesis, atherosclerotic plaque formation is a side effect of *Helicobacter cinaedi* infection, which can cause chronic inflammation and lipoprotein metabolism disorder in macrophages leading to the development of atherosclerosis when translocated in the vasculature (Khan et al., 2014). This microorganism is a natural symbiont of the gastrointestinal tract of some mammals (Gebhart et al., 1989), but in humans it is considered a conditional pathogen such that in the case of immune system malfunction, the bacterium can enter the bloodstream and cause bacteremia. Clinically, this microorganism is found in atherosclerotic plaques of people who died of atherosclerosis (Khan et al., 2012). Furthermore, it has been experimentally shown that oral infection of Apolipoprotein E (ApoE)-deficient mice with *H. cinaedi* results in the more frequent development of atherosclerotic plaques in the lumen of vessels compared to uninfected ApoE-deficient mice (Khan et al., 2014). Macrophage cells play a key role in the progression of atherosclerosis, since the atherosclerotic plaque itself is a nidus of inflammation that attracts various leukocytes. Due to changes in lipid metabolism, macrophages in the area of the developing plaque acquire the so-called “foam cell” phenotype, one of the main markers of the beginning of atherosclerotic vascular lesions. Further increase of atherosclerotic plaque in size occurs mainly due to the attraction of new macrophage cells to the center of inflammation, which can also accumulate lipids (Libby et al., 2019).

To date, *H. cinaedi* is a poorly studied pathogen, but more and more human diseases associated with this bacterium are documented each year (van der Ven et al., 1996; Lewis et al., 2007; Nishida et al., 2016). One of the known *H. cinaedi*-associated diseases is bacteremia (Fox-Lewis et al., 2020; Fujita et al., 2021), but other pathologies such as cellulitis (Lenskaya et al., 2022), ovarian abscess (Sato et al., 2019), and vertebral osteomyelitis (Hase et al., 2018) are also reported. Among patients with *H. cinaedi*-associated pathologies, there are different age groups, including young people without immunodeficiencies.

## 2 *Helicobacter cinaedi* bacterium characterization and pathogenic properties

**Figure 1:**
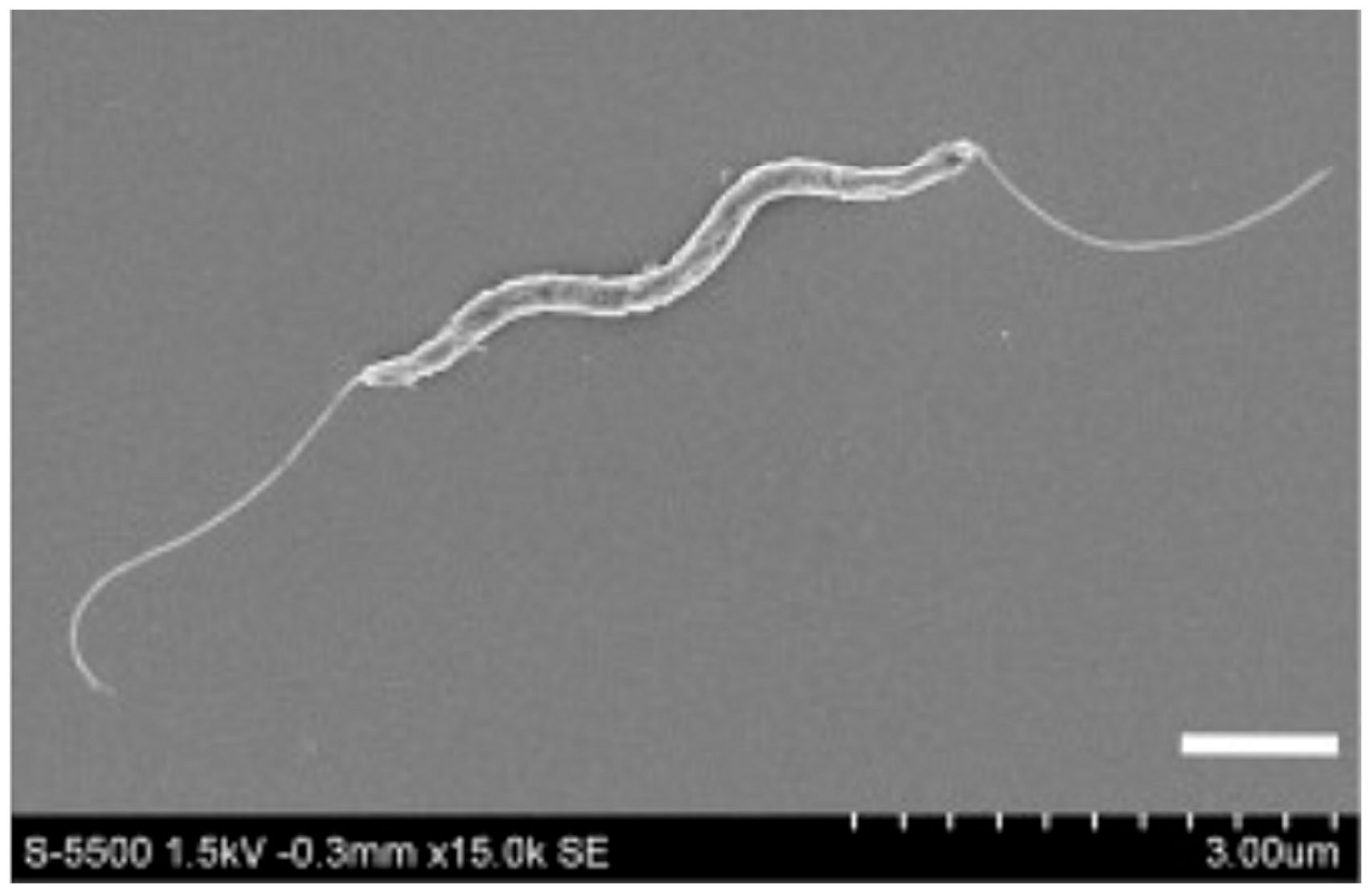
A scanning electron microscope photograph of *Helicobacter cinaedi* bacterium; magnification 15,000x. Reprinted with permission from Clinical and Bacteriological Characteristics of *Helicobacter Cinaedi* Infection by Kawamura et al. (2014), licensed under Creative Commons CC-BY-NC-ND license.

*Helicobacter cinaedi* is a spiral-shaped Gram-negative bacterium (family *Helicobacteriaceae*, order *Campylobacterales*, class *Epsilonproteobacteria*). The bacterium has an elongated spiral shape with bipolar flagella (Figure 1), but as the culture ages, it changes its shape to coccoid, folding like a tangle. The cell size varies from 0.2 to 1.2 μm in width and from 1.5 to 10 μm in length and under a light microscope at ×1,000 magnification *Helicobacter cinaedi* looks filamentous (Kawamura et al., 2014). For humans, the bacterium is conditionally pathogenic, which corresponds to WHO risk group 2 (HSE, n.d.).

To date, there is a genome annotation for the strain *H. cinaedi* BAA-847 (CDC DO148) (Miyoshi-Akiyama et al., 2012). The genome of this strain is represented by a circular chromosome 2,240,130 bp in length with an average GC content of 38.34%. The chromosome contains 2,322 protein-coding genes, 40 tRNA genes for all amino acids, two rrn operons, and three prophage-like elements.

It is known that a number of *H. cinaedi* strains naturally contain the plasmids (Kiehlbauch et al., 1995). For strain PAGU611 isolated from a patient with bacteremia, the genomic sequence of plasmid pHci1 was identified with a size of 23,054 bp (Goto et al., 2012). In a recent paper, Yasuhiro Gotoh and coworkers report seven plasmids found in strain T36 obtained from hamster intestines (Taniguchi et al., 2014; Gotoh et al., 2022). The authors of the article also mention the plasmid pD7095-1 found in strain D7095, but the article does not provide a sequence reference for this plasmid. Regarding pHci1, this paper reports that no genes encoding rep proteins were found when the sequence of the plasmid was analyzed, and the sequence of the plasmid is very similar to part of the sequences of ATCC strains BAA-847 and P01D0000. Based on these data, the authors express doubt as to whether pHci1 is actually a plasmid, or whether it is a section of the genome of the chromosome of the bacterium itself. To date, we have been unable to find any further information in the literature about native plasmids of *H. cinaedi* or attempts to transform this species with genetically engineered plasmids.

In terms of nutrition *Helicobacter* genus members are chemoorganotrophs, i.e., they obtain all necessary nutrients and energy by biological oxidation of organic substances. For better nitrogen assimilation microorganisms of this group produce urease, and in terms of oxygen demand, they are classified as microaerophiles given their limited ability for oxygen respiration. The most optimal conditions for cultivation of *H. cinaedi* is a gas mixture of 6% O_2_, 7% H_2_, 7% CO_2_, and 80% N_2_, but the minimum sufficient gas levels are O_2_ at 3–10% and СО_2_ at 3–15%. Oxygen content of more than 15% is toxic to this microorganism, and the presence of hydrogen in the gas mixture, on the contrary, favors the growth of the bacterium. Thus, cultivation of *H. cinaedi* requires a special atmosphere containing a reduced amount of oxygen (3–15%) and an increased amount of carbon dioxide (3–10%) compared to atmospheric air (Kawamura et al., 2014). It is reasonable that approximately the same oxygen and carbon dioxide content is characteristic of the human gastrointestinal tract (Keeton et al., 2023), where representatives of the genus *Helicobacter* are often found.

For cultivation of *H. cinaedi*, it is recommended to use modified Levinthal medium (Tomida et al., 2013) or Columbian medium^1^ at an ambient temperature of 37°C. ATCC recommend using the following mediums: ATCC Medium 1115: Brucella albimi broth; ATCC Medium 260: Trypticase soy agar/broth with defibrinated sheep blood, and they also specify that the bacterium requires higher humidity when cultured on solid medium.^2^

## 3 The history of research on the bacterium *Helicobacter cinaedi*

The bacterium was first isolated in 1984 from human rectal cultures and named “Campylobacter-like organism type-1” (CLO-1) (Fennell et al., 1984). The authors pointed out that unlike other known *Campylobacter* species, CLO-1 was slow growing, had an unusual colony morphology, and did not grow at 25°C. In 1985, the same research group showed that there were two genetic groups within the CLO-1 type, named CLO-1a and CLO-1b, with DNA–DNA hybridization values of 42–51%, and the name “*Campylobacter cinaedi*” was proposed for this microorganism (Totten et al., 1985). Later, based on bacterial DNA hybridization experiments and immunotyping of the genus *Campylobacter and* related taxa, the bacterium was assigned to the genus *Helicobacter* (Vandamme et al., 1991). Thus, in publications since 1991, the bacterium is named *Helicobacter cinaedi*.

A recent study shows the position of *H. cinaedi* within the genus *Helicobacter* in terms of modern systematics (Smet et al., 2018). It should be noted that representatives of this genus can be conditionally divided into gastric species, which exclusively colonize the stomach, and enterohepatic species, which colonize the liver or intestinal tract of animals (Solnick and Schauer, 2001). *Helicobacter cinaedi* is an enterohepatic member of the genus *Helicobacter*, whereas *H. pylori*, the best known member of the genus, belongs to the gastric species.

Back in the 1980s, when *H. cinaedi* was first discovered, it was realized that this species was characterized by genetic variation (the first variations described were the so-called CLO-1a and CLO-1b) (Totten et al., 1985). Currently, many strains of *H. cinaedi* are known, and another recent study shows that this microorganism appears to be a human-adapted variation of the *Helicobacter cinaedi/canicola/'magdeburgensis'* group (Gotoh et al., 2022). The authors of the article provide a detailed scheme of phylogenetic relationships between 67 strains of this group, which allows not only to trace the evolutionary relationship between different strains of *H. cinaedi*, but also to look at the closest genetic variations found in the gastrointestinal tract of humans and different animal species.

Regarding the occurrence of *H. cinaedi* in animals, in 1989, 5 years after the discovery of *H. cinaedi*, the first report appeared that this microorganism appeared to be a normal representative of the gastrointestinal microflora in hamsters. All isolates of *H. cinaedi* (at that time still named *Campylobacter cinaedi*) isolated from the feces of healthy hamsters were phenotypically and protein profile similar to the human strain of *C. cinaedi* ATCC 35683 (Gebhart et al., 1989). Somewhat later, a paper appeared mentioning several strains of *H. cinaedi* derived from isolates from dogs and cats (Kiehlbauch et al., 1995). The authors of the work do not specify whether these were healthy animals or had any signs of disease.

The isolation of *H. cinaedi* from samples obtained from various animals has been repeatedly reported. In addition to hamsters, dogs, and cats, *H. cinaedi* has been reported in a number of zoo animals: red panda (*Ailurus fulgens fulgens*), Nile crocodile (*Crocodylus niloticus*), sea lion (*Zalophus californus*), brown bear (*Ursus actus*), and Amur tiger (*Panthera tigris altaica*) (Al-Soud et al., 2003). In this case, the samples were obtained as part of the screening of animal fecal microflora, and no clinical reasons for testing were indicated in the article; however, another paper reported that fecal analysis of healthy domestic rhesus macaques (*Macaca mulatta*) revealed *H. cinaedi* in 31% of animals (Fernandez et al., 2002). Healthy natural-born long-tailed macaques (*Macaca fascicularis*) were also found to have *H. cinaedi* as part of their internal microflora (Sawaswong et al., 2023). Thus, it can be assumed that in many animals, both mammals and reptiles, *H. cinaedi* is a normal representative of the internal microflora.

However, it is worth listing the publications describing the association of *H. cinaedi* with primate pathologies. In 1990, an experiment on oral infection of pig-tailed macaque (*Macaca nemestrina*) cubs with *H. cinaedi* was conducted, resulting in diarrhea and some signs of bacteremia without accompanying fever (Flores et al., 1990). More recently, there was a report of isolation of *H. cinaedi* from the intestine of a 2-year-old rhesus macaque with chronic diarrhea, colitis, and hepatitis (Fox et al., 2001) and the large intestine of a baboon (*Papio anubis*) with pancreatic islet amyloidosis and hepatitis (García et al., 2006). In addition, there are repeated references to experimental infection of mice with *H. cinaedi* leading to the development of various pathologies (Khan et al., 2014; Shen et al., 2015; Taniguchi et al., 2017), and in another case, the bacterium was detected in the feces of a puppy with bloody diarrhea (Misawa et al., 2002).

The distribution of the bacterium in different animal groups is of interest to researchers primarily in the context of animal-to-human transmission of *H. cinaedi*. When it was discovered in the 1980s that this bacterium was part of the normal microflora of hamsters, the authors suggested that animals could be a natural reservoir of *H. cinaedi* from which the bacterium could be transmitted to humans (Gebhart et al., 1989). There has been a case report of the pathogen causing meningitis in a newborn infant whose mother kept hamsters as pets during the first two trimesters of pregnancy (Orlicek et al., 1993), but without direct evidence of animal-to-human transmission. One study examined the ability of hamster- and dog-derived *H. cinaedi* isolates to adhere, invade, and translocate through polarized human intestinal epithelial Caco-2 cells *in vitro* (Taniguchi et al., 2016). It turned out that the bacterium does have pathogenic potential in human epithelial cells, but at present there are still no documented cases of human infection with this bacterium from animals.

**Figure 2:**
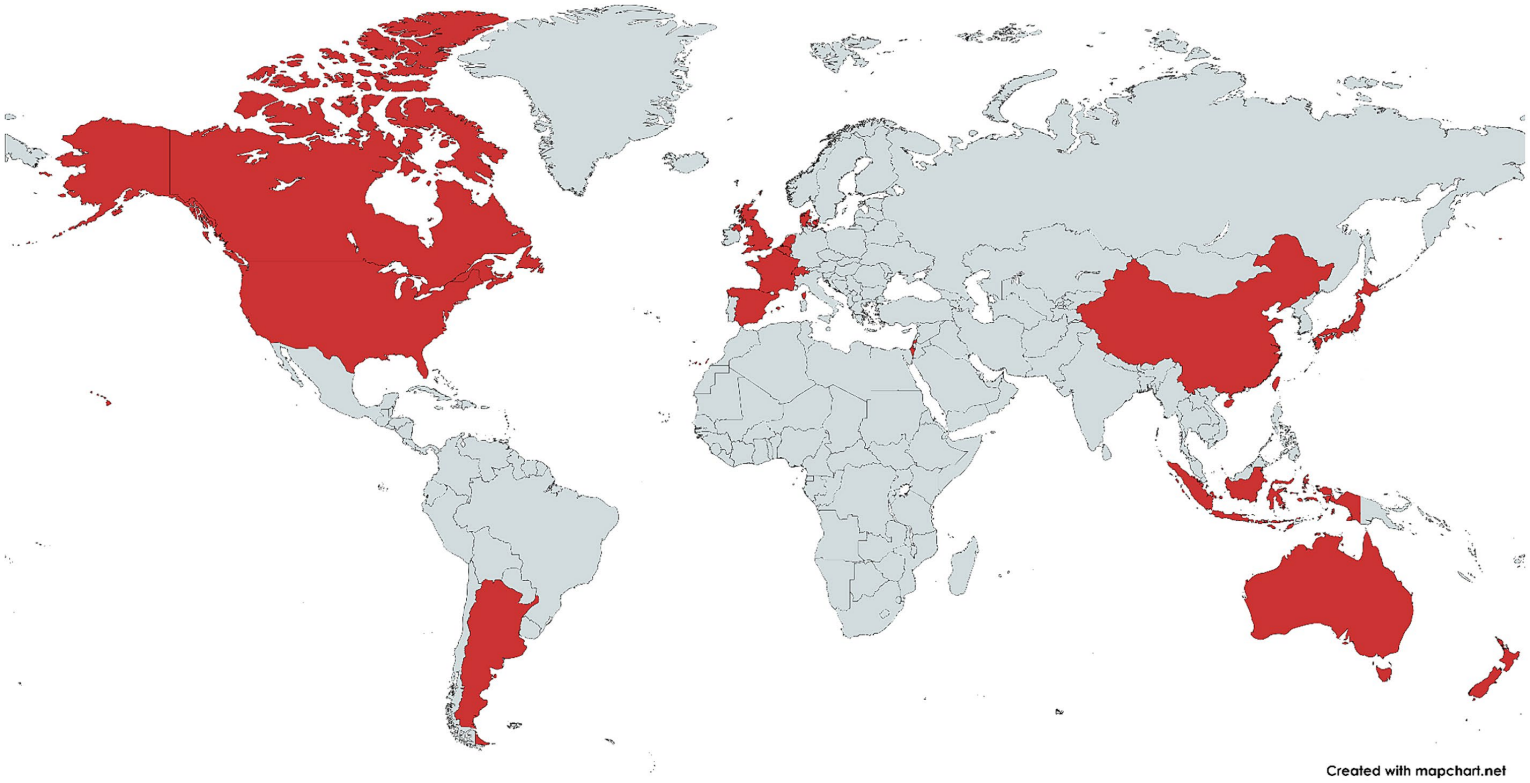
Countries where *Helicobacter cinaedi*-related diseases have been reported (highlighted in red). The image was obtained using the mapchart.net service based on a search at https://www.ncbi.nlm.nih.gov/pmc/ using the keywords “*Helicobacter cinaedi*.”

To date, there is no precise data on the distribution of *H. cinaedi* in the human population. However, clinical reports provide an indication of the occurrence of this bacterium in different countries of the world (Figure 2). Japan is the leader in reported *H. cinaedi* cases, and a number of clinical cases associated with this bacterium have been reported in the United States and France. The remaining countries have only a few reports of *H. cinaedi* (source: https://www.ncbi.nlm.nih.gov/pmc/, keyword search “*Helicobacter cinaedi*”). Nevertheless, the fact that the bacterium has been recorded in different regions of the globe suggests that this pathogen may be widespread. In addition, publications have repeatedly emphasized that *H. cinaedi* is difficult to detect by standard methods of analysis because the bacterium is characterized by slow growth and requirements for atmospheric composition (Kawamura et al., 2014; Matsumoto et al., 2017; Nukui et al., 2020). Thus, in some cases, the presence of *H. cinaedi* in humans may not have been detected due to the specific culturing conditions required for the bacterium. In recent years, new, more sensitive methods of analysis have become available, the use of which may increase the frequency of detection of the bacterium in patient samples (Hase et al., 2018; Narita et al., 2018; Nakamura et al., 2022).

## 4 Pathologies associated with the bacterium *Helicobacter cinaedi*

The bacterium was first detected in Seattle, United States, in the analysis of rectal cultures from a homosexual male patient with symptoms of intestinal distress (Fennell et al., 1984). In the following years, there were a number of clinical reports emphasizing that *H. cinaedi* was found mainly in the samples of homo- or bisexual men with immunodeficiencies, due to which the bacterium was considered to be HIV-associated (Cimolai et al., 1987; Ng et al., 1987; Grayson et al., 1989; Kemper et al., 1993; Burman et al., 1995). Later, the bacterium was found in other patient groups, including women and children (Vandamme et al., 1990; Skirrow et al., 1993). Currently, both cases of *H. cinaedi* infection against a background of immunodeficiencies (Saito et al., 2022; Roupie et al., 2023) and in immunocompetent patients have been reported (Akiyama et al., 2021; Toyoshima et al., 2022).

Table 1 summarizes examples of *H. cinaedi* associated diseases documented in the last 5 years. It is worth noting that in all the cases cited, *H. cinaedi* was identified as the cause of the disease, followed by treatment (mainly antibiotic therapy) for this diagnosis. In most cases, the therapy led to recovery of patients.

**Table 1:** Published examples of diseases associated with *Helicobacter cinaedi.*

Patient no.	Age	Disease	Immunodeficiency (+/−)	Country	Source
1	72	Infected aortic aneurysm	+	Japan	Saito et al. (2022)
2	64		+	Japan	
3	88		+	Japan	
4	83		+	Japan	
5	72		+	Japan	Matsuoka et al. (2021)
6	80		-	Japan	Toyoshima et al. (2022)
7	77		-	Japan	Matsuo et al. (2020)
8	85		-	Japan	
9	72		-	Japan	
10	61	Bacteremia	+	Japan	Fujita et al. (2021)
11	74		+	Japan	Nagase et al. (2021)
12	86		+	Japan	Suzuki S. et al. (2019)
13	63		+	Japan	Fujita et al. (2018)
14	44		+	Denmark	Rasmussen et al. (2021)
15	31		-	New Zealand, possibly also Indonesia, Singapore	Fox-Lewis et al. (2020)
16	74		-	Japan	Yasuda et al. (2019)
17	74		-	Japan	Suzuki T. et al. (2019)
18	76		-	Argentina	Togneri et al. (2019)
19	0 (37-day-old infant)		-	Argentina	
20	72		+	Japan	Takenaka et al. (2021)
21	43		-	France	Beauruelle et al. (2019)
22	61	Cellulitis	+	France	Roupie et al. (2023)
23	23		+	United States	Lenskaya et al. (2022)
24	24		+	Japan	Inoue et al. (2020)
25	72	Infected subdural hematoma	-	Japan	Akiyama et al. (2021)
26	11	Possible chronic cholangitis	+	United States	Fettinger et al. (2021)
27	38	Ovarian abscess	-	Japan	Sato et al. (2019)
28	50	Thyroid abscess	-	Japan	Takehara et al. (2019)
29	32	Infection of the prosthetic joint	+	France	Kedra et al. (2018)
30	65	Vertebral osteomyelitis	-	Japan	Hase et al. (2018)

Since this species is still poorly studied, only three virulence factors of *H. cinaedi* have been described in the literature: cytolethal distending toxin (Cdt) (Taylor et al., 2003), alkyl hydroperoxide reductase (AhpC) (Charoenlap et al., 2012), and *Helicobacter cinaedi* autotransporter protein (HcaA) (Aoki et al., 2023). Cdt is a lethal toxin that induces apoptosis and cell cycle arrest in the host cell, while AhpC is an enzyme that converts various alkyl hydroperoxides into the corresponding alcohols and promotes the survival of the bacterium inside the host cell by converting hydrogen peroxide into water (defense against oxidative stress). HcaA is an autotransporter, a type V secretion system protein that promotes adhesion to the host cells. In addition to the above three factors, a type VI secretion system (T6SS) has been found in *H. cinaedi* (Goto et al., 2012). For the closely related species *H. hepaticus*, an association between the protein of this secretion system VgrG1 and the occurrence of colitis has been described (Bartonickova et al., 2013). Therefore, it can be assumed that in *H. cinaedi* T6SS plays a similar role in the pathogenesis of gastrointestinal diseases, but no direct evidence has been published yet.

Recently, a paper has been published reporting computer modeling of a vaccine against *H. cinaedi* (Ismail et al., 2022). This is the first project of a preventive strategy whose action is directed specifically at this microorganism. Today, treatment of patients infected with *H. cinaedi* is based on antibiotic therapy (Lenskaya et al., 2022; Saito et al., 2022; Roupie et al., 2023).

## 5 Association of the bacterium *Helicobacter cinaedi* with the development of atherosclerosis

### 5.1 Foam cell formation

Lipoproteins are involved in the transport of cholesterol and its uptake by cells: high-density lipoproteins (HDL) participate in the transport of cholesterol from the bloodstream to the liver and other depots, while low-density lipoproteins (LDL), on the contrary, transport cholesterol from the depot to the bloodstream (Feingold, 2021). Foam cell formation occurs when the macrophage endoplasmic network malfunctions due to the accumulation of excessive amounts of free cholesterol inside the cell (Gui et al., 2022).

Since atherosclerotic lesions are initiated by excessive amounts of LDL (Libby et al., 2019), proinflammatory macrophages that end up in the lesion focus tend to engulf LDL to restore normal lipoprotein balance. The cellular system of receptors and enzymes is reorganized for this task: the number of receptors and enzymes promoting lipoprotein uptake from outside increases and the number of receptors and enzymes associated with lipoprotein removal from the cell decreases (Chistiakov et al., 2017; Gui et al., 2022). The most frequent and first change is an increase in the number of acceptor receptors, which have affinity for oxidized low-density lipoprotein (oxLDL): SR-A1, CD36 and LOX-1, lectin-like oxLDL receptor-1. It is important that only modified LDL (oxLDL) can serve as an external stimulus to increase the number of these receptors on the cell surface (Chistiakov et al., 2016; Orekhov et al., 2020).

**Figure 3:**
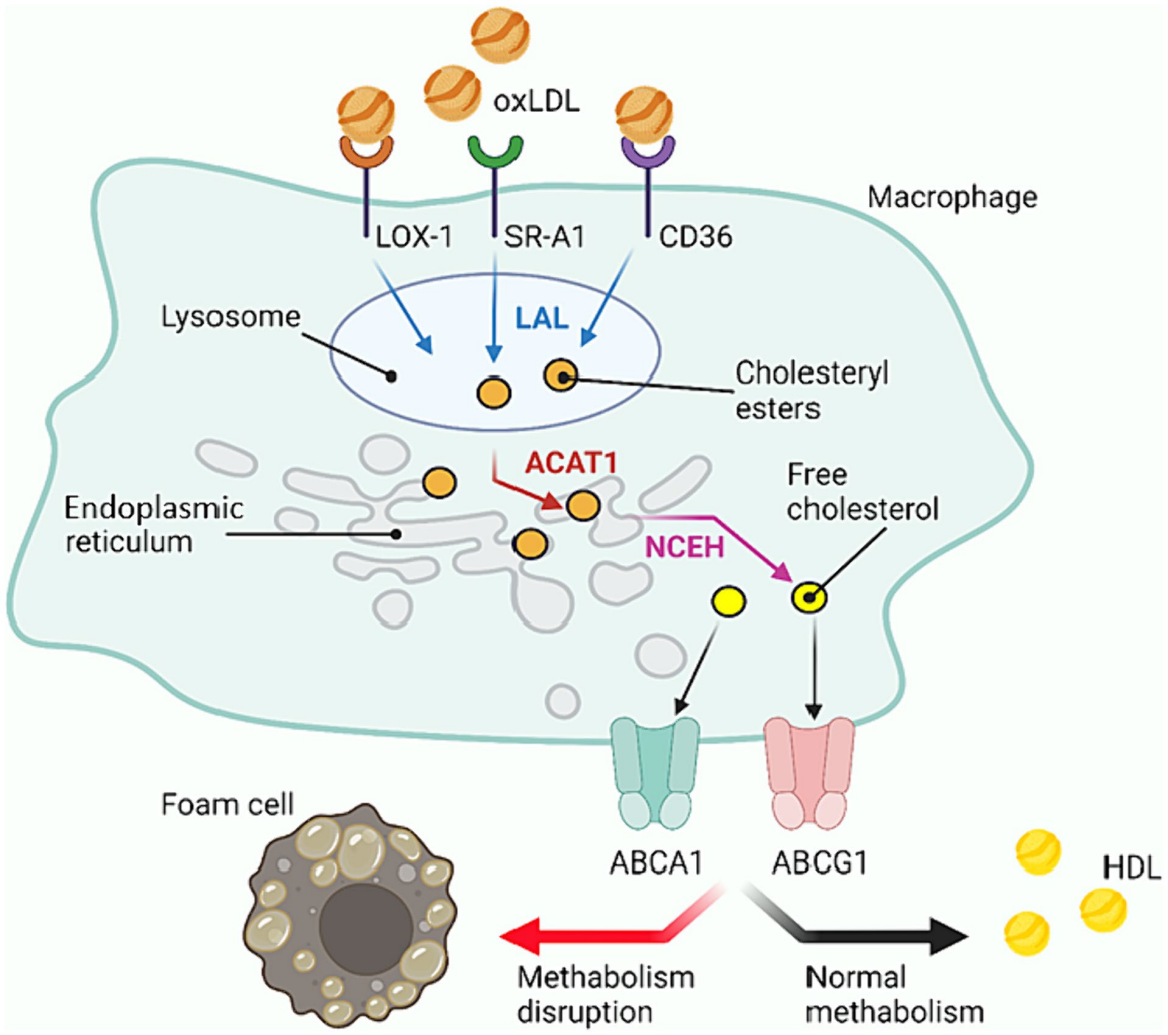
Cholesterol metabolism within macrophage cells. oxLDL, Oxidized low density lipoprotein; HDL, High density lipoprotein; LOX-1, Lectin-like oxidized low-density lipoprotein receptor-1; SR, Scavenger receptor (in the scheme SR-A1); LAL, Lysosomal acid lipase; ER, Endoplasmic reticulum; ACAT1, Cholesterol acyltransferase-1; NCEH, Neutral cholesteryl ester hydrolase; ABC, ATP-binding cassette transporters (ABCA1 and ABCG1 in the scheme). Created with BioRender.com.

The normal cycle of cholesterol metabolism inside macrophage cells is as follows (Figure 3). After LDL uptake, the cholesterol within them is converted to cholesteryl esters under the action of lysosomal acid lipase (LAL) and acetyl-CoA acetyltransferase (ACAT1). These esters are deposited in the endoplasmic reticulum (ER) (Liu et al., 2022). Then neutral cholesteryl ester hydrolase (NCEH) hydrolyzes the cholesteryl esters, releasing free cholesterol, which is transported outward through the membrane cholesterol carrier system (Kume et al., 2000). In this way, macrophage cells recycle LDL. The cholesterol released in this process can further be incorporated into HDL, which helps to maintain lipoprotein balance in the body. The foam cell formation occurs due to “congestion” at the stage when cholesterol esters are processed within the cell, i.e., the cellular system cannot deal with the volume of cholesterol esters passing through, resulting in the accumulation of excessive amounts of cholesterol esters in the ESR. All factors contributing to the accumulation of cholesterol esters inside the cells can lead to such a disorder: increased number of receptors (greater capture of particles with cholesterol esters from outside), decreased pumping of free cholesterol out of the cells (more particles remain in the cells), failure of enzymes, etc. (Chistiakov et al., 2017).

The foam cell formation may be caused by exposure to bacterial antigens and products of intestinal microbiota. For example, one study found that nine species of bacteria: *Acinetobacter baumannii*, *Escherichia coli*, *Klebsiella pneumoniae*, *Pseudomonas. aeruginosa*, *Pseudomonas. diminuta*, *Proteus. vulgaris*, *Staphylococcus aureus*, *Staphylococcus epidermidis*, and *Streptococcus salivarius* lead to the formation of lipid granules in macrophages by stimulating Toll-like receptors (TLRs) (Nicolaou et al., 2012). The tuberculosis pathogen *M. tuberculosis* uses lipids to build its cell wall and as a source of energy (Muñoz-Elías et al., 2006; Lee et al., 2013), so by accumulating lipids it is able to influence the lipid metabolism of the host cell by stimulating the expression of relevant genes (Nazarova et al., 2017, 2019; Agarwal et al., 2021). Some products of intestinal microbiota metabolism: trimethylamine-N-oxide (TMAO), indoxyl sulfate, p-cresol, and short-chain fatty acids also contribute to the formation of foam cells by stimulating scavenger receptor formation and inhibiting cholesterol efflux mechanisms from macrophage cells (Chistiakov et al., 2017; Chaves et al., 2021). The above-mentioned work of Khan’s group also demonstrates the foam cell formation as a result of contact with the bacterium *H. cinaedi* (Khan et al., 2014).

### 5.2 Foam cell role in the development of atherosclerosis

The name “atherosclerosis” is derived from the Greek athērē “groat,” “porridge,” and sklērōsis “hardening,” which reflects the process of accumulation of fibrous lipoprotein material in the lumen of blood vessels that occurs in this disease. Due to the disruption of normal lipid and protein metabolism, fatty and/or fibrous lipoprotein fractions begin to deposit in the arterial intima. This is how atherosclerotic plaque (otherwise known as atheromatous plaque, cholesterol plaque or atheroma) is formed. Over time, the plaque may change consistency, become more fibrous and begin to accumulate mineral calcium, which leads to even greater thickening of the plaque. The growth of atheromatous plaque leads to narrowing of the blood vessel lumen, impaired blood flow, and a high risk of thrombus formation, which completely occludes the lumen resulting in acute ischemia (Libby et al., 2019).

**Figure 4:**
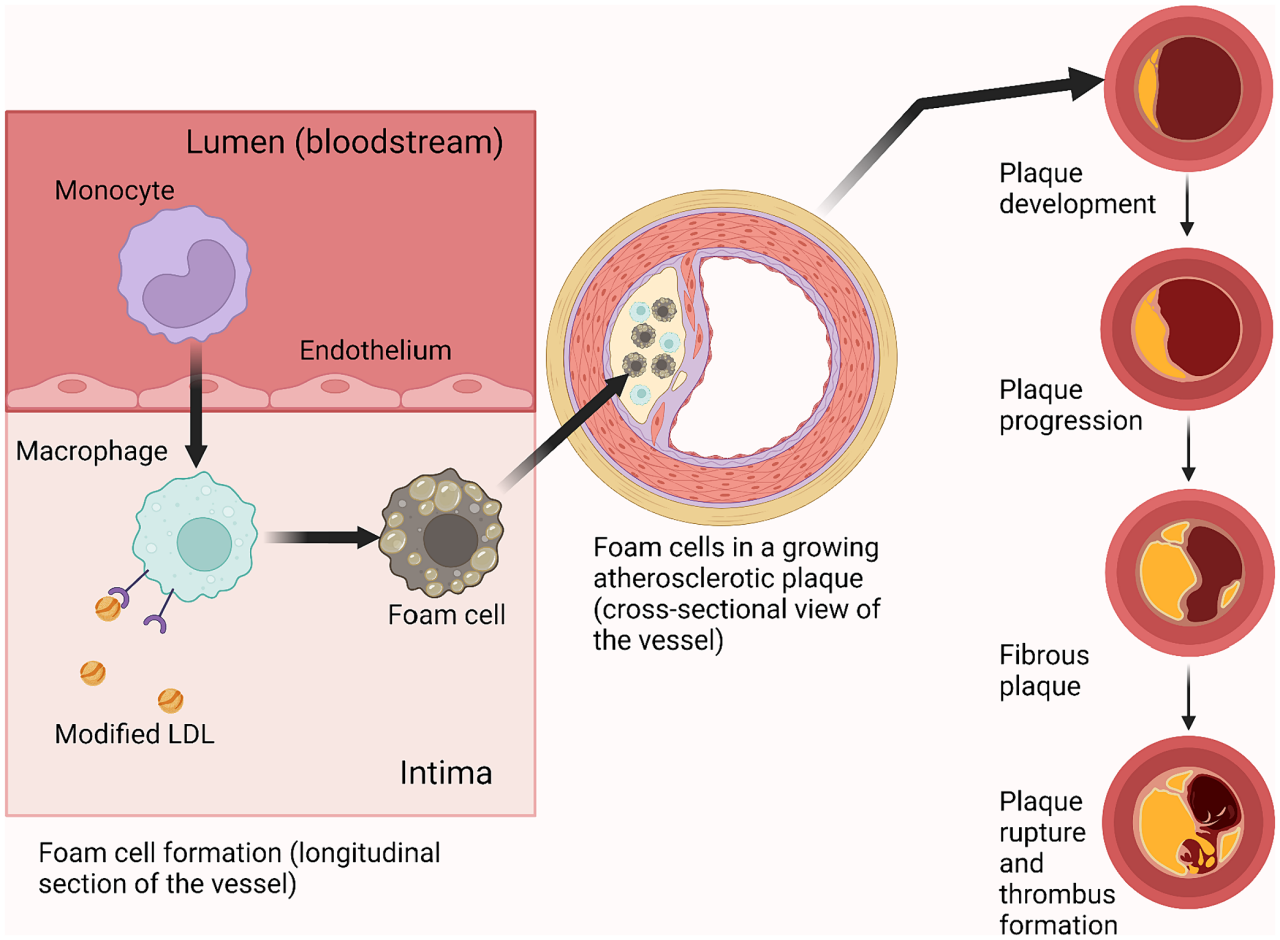
Foam cell role in atherosclerotic plaque progression. Created with BioRender.com.

Macrophage cells play a significant role in the progression of atherosclerosis, and it is the foam cells (alive or undergoing apoptosis) that form the basis of a growing cholesterol plaque (Figure 4) (Wanschel et al., 2013; Libby et al., 2019). The course of atherosclerosis is always accompanied by an inflammatory reaction, attracting monocytes to the lesion, which then differentiate into macrophages. Two types of macrophages can form as a result of differentiation: M1 (pro-inflammatory) and M2 (anti-inflammatory) (Yunna et al., 2020). The formation of M1-type macrophages occurs under the influence of GM-CSF and IFN-ɣ (Ushach and Zlotnik, 2016; Lu et al., 2019), while the formation of M2-type macrophages is promoted by IL-4 and IL-13 (Italiani and Boraschi, 2014; Genin et al., 2015). Both types remain in the lesion and produce adhesion factors, contributing to atheroma formation.

At the beginning and throughout most of the development of the disease, the atheroma grows radially in an abluminal direction (forming a ring around the perimeter of the vessel wall), while maintaining the caliber of the arterial lumen. Then, upon reaching a certain size, the lesion begins to invade the vessel lumen, obstructing normal blood flow which, under conditions of exercise or stress when the myocardium has an increased oxygen demand, can lead to complications such as coronary heart disease and angina pectoris (Libby et al., 2019). The most serious complication of atherosclerosis is thrombus formation due to rupture of an atherosclerotic plaque, which can lead to myocardial infarction (Ruiz et al., 2016).

### 5.3 *Helicobacter cinaedi* and atherosclerosis

The specific causes leading to pathologic changes in lipoprotein metabolism, inflammation, and atherosclerosis, as well as virulence factors of *H. cinaedi*, are poorly understood, but there are studies that show the relationship between the presence of this bacterium and the progression of atherosclerosis. The most complete of them to date is the article by Khan et al. where experiments on cellular and mouse models were performed (Khan et al., 2014). In this study, they showed foam cell formation when *H. cinaedi* infected peritoneal macrophages from wild-type mice and human monocyte-derived macrophages; at the same time, *H. pylori* infection did not lead to the formation of foam cells. The authors note that in *H. cinaedi*-infected cells lipid droplets were accumulated even without the addition of LDL in the culture medium, whereas in similar experiments with *C. pneumoniae* foam cells were formed only in the presence of exogenous LDL in the culture medium (Cao et al., 2007). Analogous results (foam cell formation without the addition of exogenous LDL) were obtained by another research group when studying Cinaedi Atherosclerosis Inflammatory Protein (CAIP), a protein of *H. cinaedi* that supposedly promotes LDL accumulation in macrophages (D’Elios et al., 2017), but there were no further publications investigating CAIP.

In addition to experiments on foam cell formation, in the study by Khan et al., it was shown that after oral infection with *H. cinaedi* the number and size of atherosclerotic plaques in the aortic sinus of mice with ApoE gene defect significantly increases compared to control uninfected mice. In the aortic tissues of infected mice, a significant increase in gene expression of inducible nitric oxide synthase, interleukin-1β, Toll-like receptor 4, C-C motif chemokine 2, and intercellular adhesion molecule-1 was observed. Also in this study, they showed that during *H. cinaedi* infection, the level of cholesterol efflux-associated protein ABCG1 was reduced in mouse peritoneal macrophages and THP-1 monocytic macrophages. Based on these results, the authors suggest a possible *H. cinaedi*-associated mechanism of atherosclerosis. Probably, the bacterium can translocate to the vascular tissue after oral infection and induce inflammatory processes leading to the development of atherosclerosis. Foam cell formation may be associated with the ability of *H. cinaedi* to downregulate ABCG1 in macrophages.

In the other clinical report made by the Khan group *H. cinaedi* antigen was detected in all postmortem samples of patients’ atherosclerotic plaques and colocalized with macrophages (Khan et al., 2012). These data suggest that *H. cinaedi* may contribute to the course or even the onset of atherosclerotic vascular lesions in humans and mammals.

Another study on finding *H. cinaedi* in atherosclerotic plaques was conducted in Turkey in 2021. 129 samples of atherosclerotic plaques from patients diagnosed with valvular heart disease due to atherosclerosis were analyzed using a nested-polymerase chain reaction. A control group of 146 patients with non-atherosclerotic post-stenotic dilatation also participated in the study. *Helicobacter cinaedi* DNA was detected only in the group of patients with atherosclerosis, in six samples out of 129 (Sarp et al., 2021). Statistical analysis showed that in this study, the presence of *H. cinaedi* was not an independent variable for the risk of atherosclerosis. However, since this work used other methods of analysis, its results cannot be compared with those of Japanese colleagues.

A recent study by Horii et al. (2023) reports the detection of *H. cinaedi* DNA in atherosclerotic abdominal aortic aneurysmal walls. The authors note that *H. cinaedi* DNA was detected exclusively in aneurysmal walls and was not detected in non-aneurysmal arterial walls. This is the first report of the detection of this bacterium in the walls of a clinically non-infectious abdominal aortic aneurysm. In addition, there are many reports of infected aortic aneurysms initiated by the pathogenic action of this microorganism (Table 1). It is possible that *H. cinaedi*-associated mechanisms of atherosclerosis and aortic aneurysm are based on the same inflammatory processes caused by this pathogen in the walls of blood vessels. To date, this issue remains incompletely clarified and requires additional research.

## 6 Discussion

In the early years after its discovery, *H. cinaedi* was considered to be one of the HIV-associated infections (Cimolai et al., 1987; Ng et al., 1987; Grayson et al., 1989; Kemper et al., 1993; Burman et al., 1995), but nowadays there are many reports that this microorganism has a more diverse range of associated pathologies, and the geography of recorded clinical cases and patient age categories are also becoming increasingly diverse (Vandamme et al., 1990; Skirrow et al., 1993). However, there is still a relatively small number of studies devoted to the properties of this bacterium and its distribution in the human population. This is a significant problem for assessing the real pathogenic potential of *H. cinaedi*.

Cardiovascular disease is an extremely important health problem worldwide, claiming about 18 million lives annually (WHO website: https://www.who.int/health-topics/cardiovascular-diseases). If *H. cinaedi* does play an important role in the development of atherosclerosis and subsequent severe CVD, it may be possible to develop preventive therapies directed against this bacterium and potentially significantly improve the development of therapies for CVD. Unfortunately, to date, a small number of studies on the association of *H. cinaedi* with atherosclerosis are known (Khan et al., 2014; Sarp et al., 2021).

The work with *H. cinaedi* and its detection in assays is difficult due to the very specific requirements of this bacterium for cultivation conditions. The bacterium requires a gas mixture of oxygen, nitrogen, hydrogen, and carbon dioxide, as well as high humidity (Kawamura et al., 2014). Thus, species-specific tests such as PCR or immunohistochemical analysis seem to be the most effective methods to detect *H. cinaedi* in biological samples (Narita et al., 2018; Nakamura et al., 2022). The inclusion of *H. cinaedi*-specific tests in clinical assays would help to obtain more information about the bacterium’s prevalence in the human population.

A very important aspect in the study of pathogenic properties of microorganisms is the knowledge of the produced pathogenicity factors. For example, many Gram-negative bacteria are equipped with secretion systems (six types in total, Type I to Type VI; Green and Mecsas, 2016; Naskar et al., 2021). *Helicobacter cinaedi* has a Type VI secretion system (Goto et al., 2012) and three pathogenicity factors: cytolethal distending toxin (Cdt) (Taylor et al., 2003), alkyl hydroperoxide reductase (AhpC) (Charoenlap et al., 2012), and *Helicobacter cinaedi* autotransporter protein (HcaA) (Aoki et al., 2023). However, to date, there is no information on how these pathogenicity factors function *in vivo* and what specific molecular mechanisms are responsible for the development of *H. cinaedi*-related pathologies. The study of the proteome of this bacterium, including proteins differentially expressed during infection of macrophages, may provide insight into what other protein factors are involved in the invasion of *H. cinaedi* into the host organism.

Finally, the question of transmission of *H. cinaedi* between humans and possibly from animals to humans has not been resolved to date. According to statistics, zoonotic origins account for about ⅔ of human diseases (Rahman et al., 2020). It is well known that *H. cinaedi* is found as part of the microflora of various animals (Al-Soud et al., 2003). Therefore, it is possible that *H. cinaedi*-associated pathologies in humans may be of zoonotic origin. To date, there has been no direct evidence of animal-to-human transmission of the bacterium, although *H. cinaedi* has been found as normal microflora in cats, dogs, and hamsters, all animals that are often kept as pets (Gebhart et al., 1989; Kiehlbauch et al., 1995). Additional studies in this area may clarify whether *H. cinaedi* is a zoonotic pathogen and provide more information on the prevalence of the bacterium and its contribution to human disease patterns.

## 7 Conclusion

*Helicobacter cinaedi* can cause disease of varying severity in humans as well as in some animals. The microorganism is found in the gastrointestinal tract of a number of mammals and apparently reptiles. No cases of human infection from animals have been reported to date, and so far *H. cinaedi* remains a poorly studied bacterial species. Nevertheless, an increasing variety of pathologies associated with *H. cinaedi* have been recorded in recent years, and the list of countries from which it has been reported is gradually expanding. Among other things, one clinical hypothesis suggests a link between *H. cinaedi* infection and foam cell formation and subsequent development of atherosclerosis. It should be noted that atherosclerosis is the main cause of the development of severe CVD and stroke, which according to the World Health Organization (WHO) is the leading cause of mortality worldwide. Thus, because of the difficulty of detection in assays and the limited amount of information on *H. cinaedi*, the role of this bacterium in the development of cardiovascular disease and in human mortality may be significantly underestimated. Further research is needed to clarify the role of this pathogen in contributing to human disease, the extent of its spread, and improving diagnostic methods for *H. cinaedi*.

## Author contributions

AV: Conceptualization, Resources, Visualization, Writing – original draft, Writing – review & editing. GA: Conceptualization, Funding acquisition, Project administration, Supervision, Writing – review & editing.

## Footnotes

^1^https://webshop.dsmz.de/de/DSM-5359.html

^2^https://www.atcc.org/products/baa-847
